# Correlation analysis between peripheral blood dendritic cell subsets and PD-1 in patients with peritoneal adenocarcinoma

**DOI:** 10.1590/1414-431X2023e13192

**Published:** 2024-02-19

**Authors:** Huihui Hu, Man Zhang

**Affiliations:** 1 Department of Clinical Laboratory, Beijing Shijitan Hospital, Capital Medical University, Beijing, China; 2 Beijing Key Laboratory of Urinary Cellular Molecular Diagnostics, Beijing, China; 3 Clinical Laboratory Medicine, Peking University Ninth School of Clinical Medicine, Beijing, China

**Keywords:** Dendritic cells, PD-1, Peripheral blood, Peritoneal adenocarcinoma, Flow cytometry

## Abstract

The aim of this study was to explore the association between differential percentages of dendritic cell (DC) subsets in peripheral blood and malignancy (grade and lymph node metastasis) of peritoneal adenocarcinoma patients and the frequencies of dendritic cell subsets in the normal controls. The peripheral blood of 30 patients with peritoneal adenocarcinoma and 12 healthy controls were collected for multicolor flow cytometry analysis. Peritoneal adenocarcinoma patients were grouped according to the malignant degree (grade and lymph node metastasis). Percentages of myeloid DCs (mDCs) and its subsets MDC1 and MDC2 in DCs were lower in peripheral blood of patients with peritoneal adenocarcinoma than in normal controls. The percentages of plasmacytoid dendritic cells (pDCs) and CD16+mDCs in DCs were higher than in normal controls. Compared with poor differentiation grade, patients with well/moderate differentiation grade had an increased percentage of CD16+mDCs. Contrary to CD16+mDCs, the percentage of MDC1 was lower in the well/moderate differentiation grade group. In patients with no lymph node metastasis, pDCs and CD16+mDCs levels were higher compared with patients with lymph node metastasis. mDCs and MDC1 levels had opposite results. pDCs were positively correlated with CD16+mDCs in peripheral blood of peritoneal patients, as was mDCs and MDC1. CD16+mDCs were negatively correlated with MDC1. The percentages of pDCs and CD16+mDCs in DCs were positively correlated with CD3+CD8+T cells, and pDCs also positively correlated with CD8+PD-1+T cells. Our results revealed that DCs subsets correlated with peritoneal adenocarcinoma malignancy. Dendritic cells play an independent role in the immune function of peritoneal adenocarcinoma.

## Introduction

Dendritic cells (DCs) act as the key coordinators of the immune response in the human body. Their main role is to control the action between tolerance and immunity (1). Dendritic cells originate from macrophages in bone marrow and act as sentinels of the immune system. DCs are antigen-presenting cells, and present antigens to T cells and B cells (2). Some classical mature lymphocyte lineage markers (CD3, CD19, CD20, CD56, and CD14) are not expressed on human DCs, while they have a high level of human leukocyte antigen - DR isotype (HLA-DR). In human blood, DCs are divided into two main subsets, myeloid DCs (mDCs) and plasmacytoid DCs (pDCs) (3). The difference between mDCS and pDCs is their CD11c expression, with mDCs presenting high expression levels of CD11c while pDCs do not (4). mDCs are highly efficient in antigen presentation (5). In human blood, mDCs can be divided into 3 subsets: MDC1 (CD1c+mDCs), MDC2 (CD141+mDCs), and CD16+mDCs.

The role of pDCs has been largely explored in autoimmune diseases (6). Circulating pDCs account for only 0.2-0.8% of the total peripheral blood mononuclear cells (PBMCs). According to reports, pDCs from myeloid progenitors and multipotent lymphoid progenitors produce I-IFN and stimulate T cells in mice (7). In the immune system, pDCs have indispensable roles in defending against pathogens, autoimmunity, and cancer. pDCs could bridge the innate and adaptive immune response. pDCs have antigen-presenting function, but lower than mDCs. While mDCs mainly uptake exogenous antigens, pDCs uptake endogenous peptides. In short, pDCs are complementary to mDCs in their antigen-presentation function (8-10).

In multiple studies of human head and neck tumors, lung cancer, and kidney cancer, tumor-related DCs has been reported to have decreased ability to take up, process, and express antigens and to stimulate the proliferation of allogeneic and homologous T cells (11-13). DC phenotypes in the tumor microenvironment are often suppressed to an immature state, while secreting a variety of soluble factors to suppress the immune response. One of the key factors in anti-tumor immunity suppression in the ovarian cancer microenvironment is that DCs are associated with B7-H1. Although these PD-1+B7-H1+DCs possess the classic DCs phenotype, they often exhibit immaturity, inhibition, and insensitivity to danger signals (14). Chaux et al. (15) found that tumor-associated DCs express low levels of co-stimulatory molecules, and immature DCs disable antigen-specific CD4+CD8+T lymphocytes and prevent immune responses by producing interleukin (IL)-10 and tumor growth factor (TGF)-β regulatory T cells.

MDC1 can stimulate initial CD4+T cells to proliferate and secrete a large amount of IL-12. In the case of MDC2, although they do not secrete large amounts of IL-12, they are able to take up dead and necrotic cells in preparation for subsequent cross-presentation of antigens to CD8+T lymphocytes (16,17). Activated pDCs can release type I interferon, which is a multipotent cytokine and not only has antiviral properties, but also plays a role in maintaining pDCs survival, mDCs differentiation, mDCs regulation of CD4+T and CD8+T lymphocyte response, and cross-antigen presentation (18,19). DCs can exert anti-tumor effects through the following mechanisms: MDC1 can secrete IL-12 and promote Th0-to-Th1 differentiation; MDC2 can secrete IL-4, promote Th0-to-Th2 differentiation, and stimulate humoral response. This mechanism can produce powerful anti-tumor effects in the short term. DCs can also secrete IL-6 and TGF to promote Th17 cell differentiation, and Th17 can induce fibroblasts and epithelial cells to secrete cytokines CXCL8, CXCL2, and GM-CSF, which can promote the production of neutrophils and macrophages (20).

Peritoneal adenocarcinoma is the most common pathologic type of peritoneal tumor. This cancer has an insidious onset, which makes diagnosis and treatment difficult (21). The clinical therapy for peritoneal adenocarcinoma is becoming multi-modal, including cytoreductive surgery (CRS) and hyperthermic intraperitoneal chemotherapy (HIPEC), but some patients still undergo curative resection and chemotherapy without clinical benefit. The use of immunosuppressive therapy in peritoneal neoplasms is rarely reported, and immune function in peritoneal adenocarcinoma has not been explored.

Programmed cell death-1 (PD-1) is a protein of the B7/CD28 family mainly expressed by activated T cells and acts as a key immune-checkpoint receptor in regulating immunosuppression (22,23). Our laboratory has reported a higher PD-1 expression in peritoneal adenocarcinoma patients' peripheral blood compared with normal controls (24). In this article, we will explore the correlation between DC subsets and the malignancy of peritoneal adenocarcinoma, because the differential percentages of DC subsets in the peripheral blood of peritoneal adenocarcinoma patients and normal controls have not been reported. We will further discuss the correlation between peripheral blood DC subsets and PD-1. The study of the specificity of DC subsets will provide new insight into the development of peritoneal adenocarcinoma and present new approaches to a promising therapy.

## Material and Methods

### Patients

Peripheral blood samples of 30 patients (age 55.2±13.1 years, female/male 12/18) with peritoneal adenocarcinoma and 12 gender- and age-matched healthy controls (age 50.9±7.5 years, female/male 5/7) were collected. Patients with peritoneal adenocarcinoma were approached for enrollment between June 2021 and July 2022 from Beijing Shijitan Hospital of the Affiliated Cancer Hospital of Capital Medical University. Adenocarcinoma was pathologically confirmed in all patients after surgery. The exclusion criteria were patients without complete clinical factors and laboratory data, patients with other types of cancer or other diseases that may influence DC subsets, and patients who received previous PD-1 immune checkpoint blockade. This study was approved by the Medical Ethics Committee, Beijing Shijitan Hospital, Capital Medical University, and was performed in accordance with the ethical standards laid down in the Declaration of Helsinki. Informed consent was obtained from all participants. The patients' details are listed in Table 1.

**Table 1:** Characteristics of peritoneal adenocarcinoma patients included in the study.

Group	Number	Age (years)	Gender (F/M)
Differentiation grade			
Low	17	54.5±10.1	7/10
Well/Moderate	13	57.6±9.5	5/8
Lymph node metastasis			
No	8	56.6±9.1	2/6
Yes	22	54.4±10.31	10/12

Data are reported as mean±SD.

### Flow cytometry

The Navios flow cytometer (Beckman Coulter, USA) was used for this experiment. Fresh venous blood samples were collected from patients and healthy donors with EDTA-coated vacutainer tubes. The analyses of DCs were performed using Duraclone IM Dendritic Cells tubes (Beckman Coulter). DCs lack lymphocytes lineage markers (CD3, CD19, CD20, CD56 and, CD14) and have a high level of HLA-DR. Based on CD11c expression, DCs were divided into mDCs and pDCs. Based on CD1c, CD141, and CD16, mDCs were divided into 3 subsets: MDC1 (CD1c+mDCs), MDC2 (CD141+mDCs), and CD16+mDCs. Fresh venous blood samples labeled with corresponding fluorochrome-conjugated non-immune isotypes were used as negative controls. The violet amine-reactive dye (Invitrogen, USA) was used to assess cell viability. After compensation setup, 200 uL of fresh venous blood was mixed with 5 uL of each antibody, vortexed at high speed for 6-8 s, and incubated for 15 min at room temperature. After incubation, each tube was mixed with 2 mL of erythrolysin (Beckman Coulter) and incubated for 15 min at room temperature. After washing with 3 mL of 1XPBS and centrifuging at 210 *g* for 5 min (20-25°C), the supernatant was aspirated and re-suspended in 500 uL of 1XPBS (containing 0.1% formaldehyde), and data was acquired on the flow cytometer. Before detection, volt and fluorescence compensation values of the flow cytometer were set. For the analyses of DCs subsets, 100,000 events were detected for each sample and the frequencies of DC subsets were calculated under DC gate. Finally, all data were analyzed by Kaluza software (Beckman Coulter).

### Statistical data analysis

The percentages of positive target cells in peripheral blood were compared and are reported as medians. Comparison between the two groups was performed with independent sample *t-*test or Mann-Whitney U nonparametric test. Pearson's correlation was used to analyze the correlation between parameters and calculate the r value and P value. Two-sided P<0.05 indicated a significant difference. Kaplan-Meier curves were used to analyze progression-free survival (PFS). P<0.05 indicated a significant difference. The data were analyzed using GraphPad Prism 5.0 software (GraphPad Software, USA).

## Results

### DC subset percentages in peripheral blood of normal controls and patients

**Figure 1:**
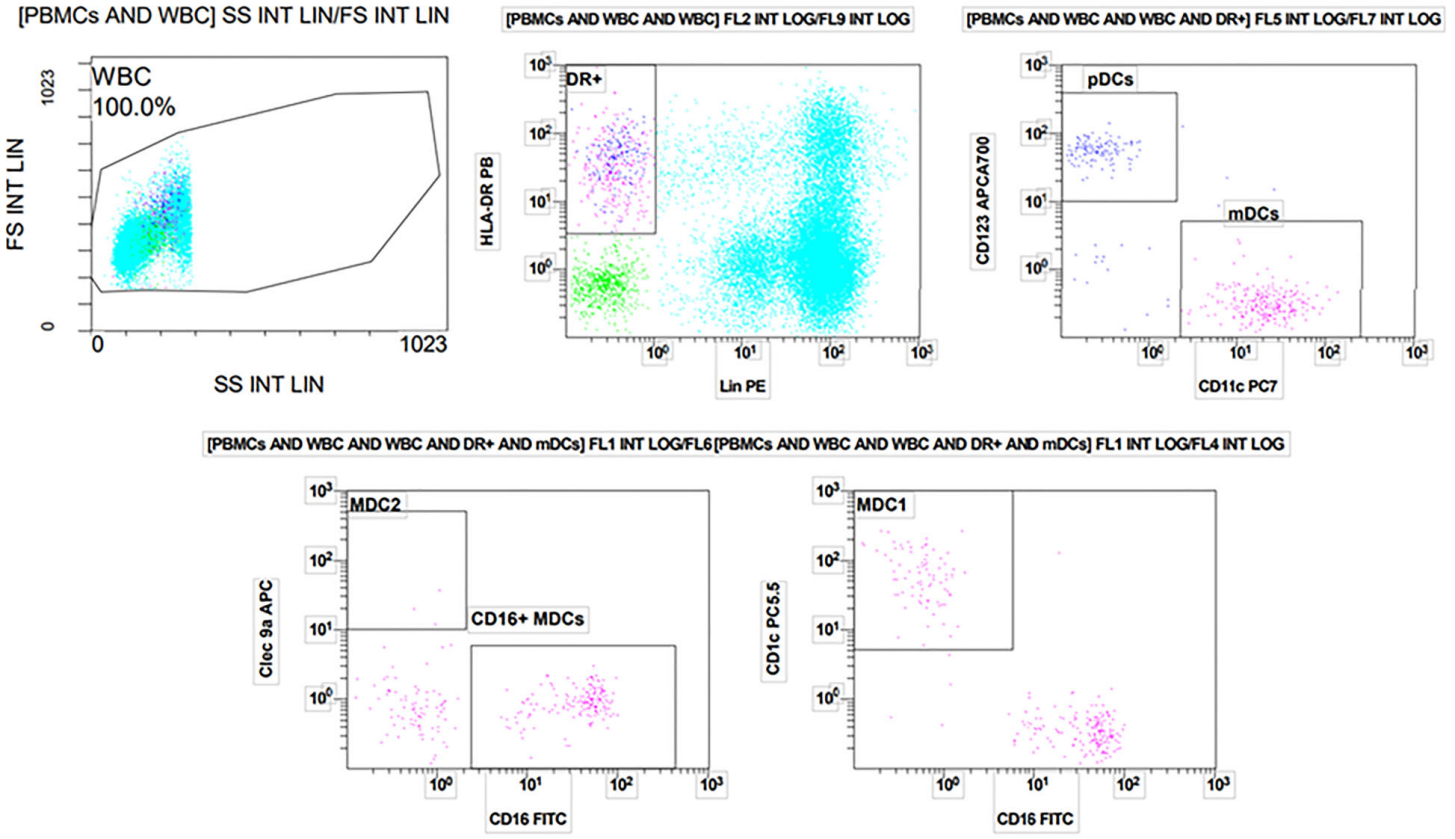
The positive percentages of dendritic cells (DCs) subsets in peripheral blood of peritoneal adenocarcinoma patients were explored by flow cytometry. DCs lack the lymphocytes lineage markers (CD3, CD19, CD20, CD56, and CD14), and have a high level of human leukocyte antigen - DR isotype (HLA-DR). DCs are divided into myeloid DCs (mDCs) and plasmacytoid DCs (pDCs); mDCs express high levels of CD11c, while pDCs do not. Based on CD1c, CD141, and CD16 expressions, mDCs can be separated into 3 subsets: MDC1 (CD1c+mDCs), MDC2 (CD141+mDCs), and CD16+mDCs. PBMCs: peripheral blood mononuclear cells; WBC: white blood cells.

**Figure 2:**
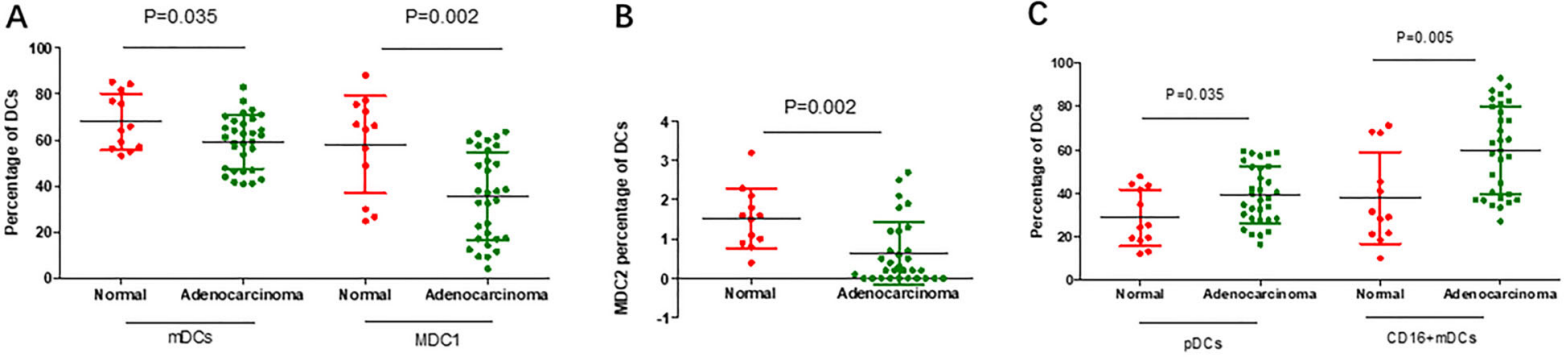
Percentages of dendritic cell (DC) subsets in peripheral blood of peritoneal adenocarcinoma patients and normal controls by flow cytometry. **A**, Myeloid DCs (mDCs) and its subset MDC1; **B**, subset MDC2; **C**, plasmacytoid DCs (pDCs) and CD16+mDCs. Data are reported as medians and interquartile range. P<0.05 was considered statistically significant. Mann-Whitney U nonparametric test.

We compared the percentages of DCs subsets (under DC gate) in 12 cases of normal controls and 30 cases of peritoneal adenocarcinoma patients by flow cytometry (Figure 1). The mDCs exhibited lower levels in the adenocarcinoma group than in normal controls (60.0 *vs* 67.9%, P=0.035, Figure 2A). In the mDCs subsets, the frequency of MDC1 cells was also significantly lower in peritoneal adenocarcinoma patients (35.6 *vs* 58.1%, P=0.002, Figure 2A). Consistent with MDC1, a significantly lower level of circulating MDC2 cells was observed in peritoneal adenocarcinoma patients compared with normal controls (0.7 *vs* 1.5%; P=0.002, Figure 2B). Because all the results were analyzed under the DC gate, the frequencies of pDCs and CD16+mDCs exhibited the opposite results. Compared with normal control, the percentages of pDCs (38.2 *vs* 31.0%; P=0.035, Figure 2C) and CD16+mDCs (59.7 *vs* 31.2%; P=0.005, Figure 2C) were higher in patients.

### Correlation of circulating DC subsets with clinicopathological features and PFS

**Figure 3:**
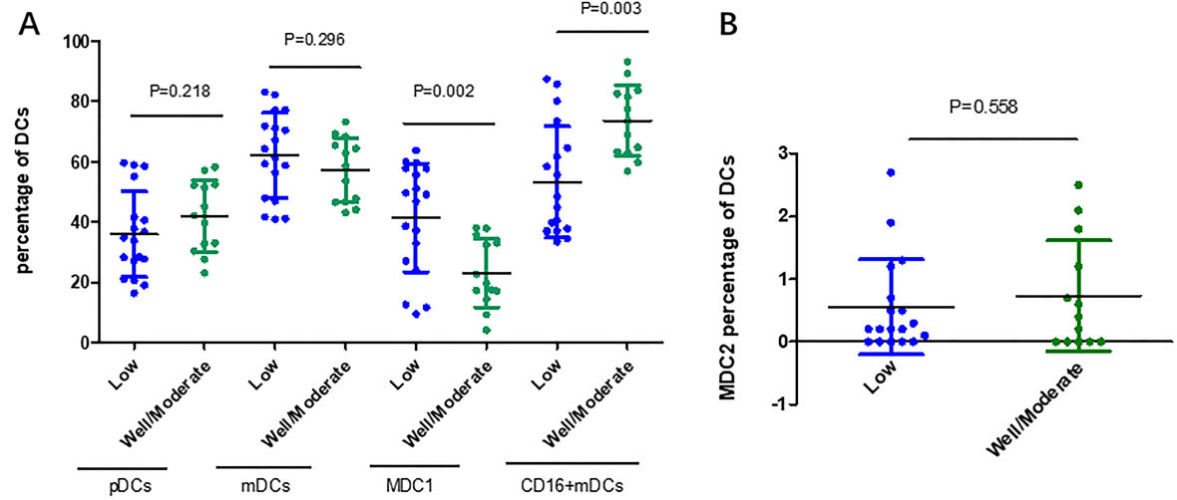
Percentages of dendritic cell (DCs) subsets and differentiation grade (low and well/moderate) of peritoneal adenocarcinoma. **A**, Plasmacytoid DCs (pDCs), myeloid DCs (mDCs), MDC1, and CD16+mDCs; **B**, MDC2. Data are reported as medians and interquartile range. Mann-Whitney U nonparametric test.

The clinical parameters investigated were differentiation grade (poor, moderate, and well) and lymph node metastasis. Compared with poor grade, the patients with high/moderate grade adenocarcinoma had higher a percentage of CD16+mDCs (73.54 *vs* 53.07%; P=0.002, Figure 3A). Contrary to CD16+mDCs, the percentage of MDC1 (22.94 *vs* 41.27%; P=0.003, Figure 3A) was lower in patients with well/moderate differentiation grade than patients with poor grade. However, the differential percentages of pDCs, mDCs, and MDC2 between the above groups was not statistically significant (P>0.05, Figure 3B).

**Figure 4:**
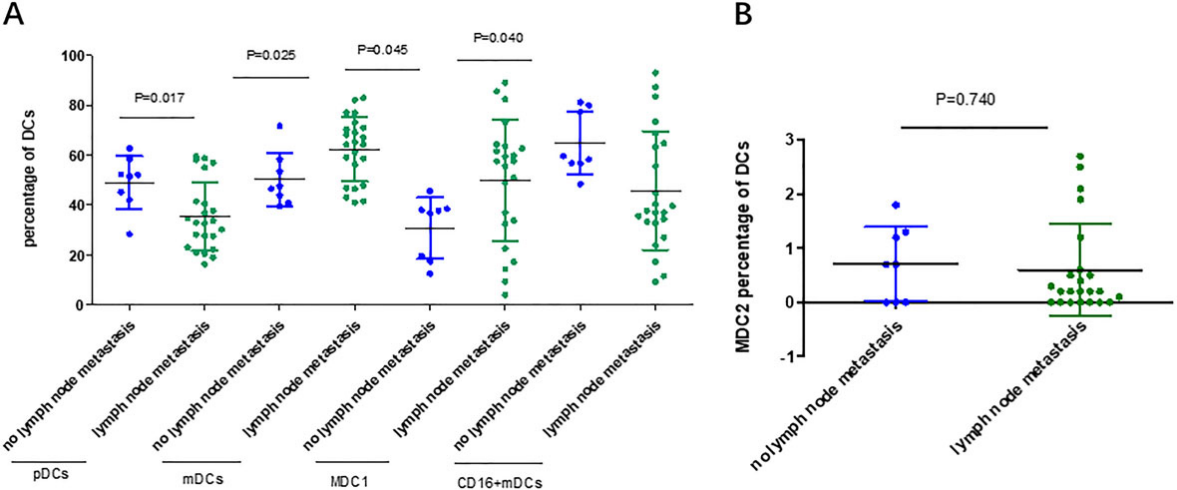
Percentages of dendritic cell (DCs) subsets in groups with and without lymph node metastasis of peritoneal adenocarcinoma. **A**, Plasmacytoid DC (pDCs), myeloid DCs (mDCs), MDC1, and CD16+mDCs; **B**, MDC2. Data are reported as medians and interquartile range. Mann-Whitney U nonparametric test.

In addition, the levels of pDCs (49.04 *vs* 35.40%, P=0.017) and CD16+mDCs (64.80 *vs* 45.69%, P=0.040) in peripheral blood of patients with no lymph node metastasis was higher compared with patients with lymph node metastasis. The levels of mDCs (50.29 *vs* 62.34%, P=0.025) and MDC1 (30.76 *vs* 49.73%; P=0.045) had opposite results (Figure 4A). The percentage of MDC2 subsets had no significant difference between the above groups (P>0.05, Figure 4B).

**Figure 5:**
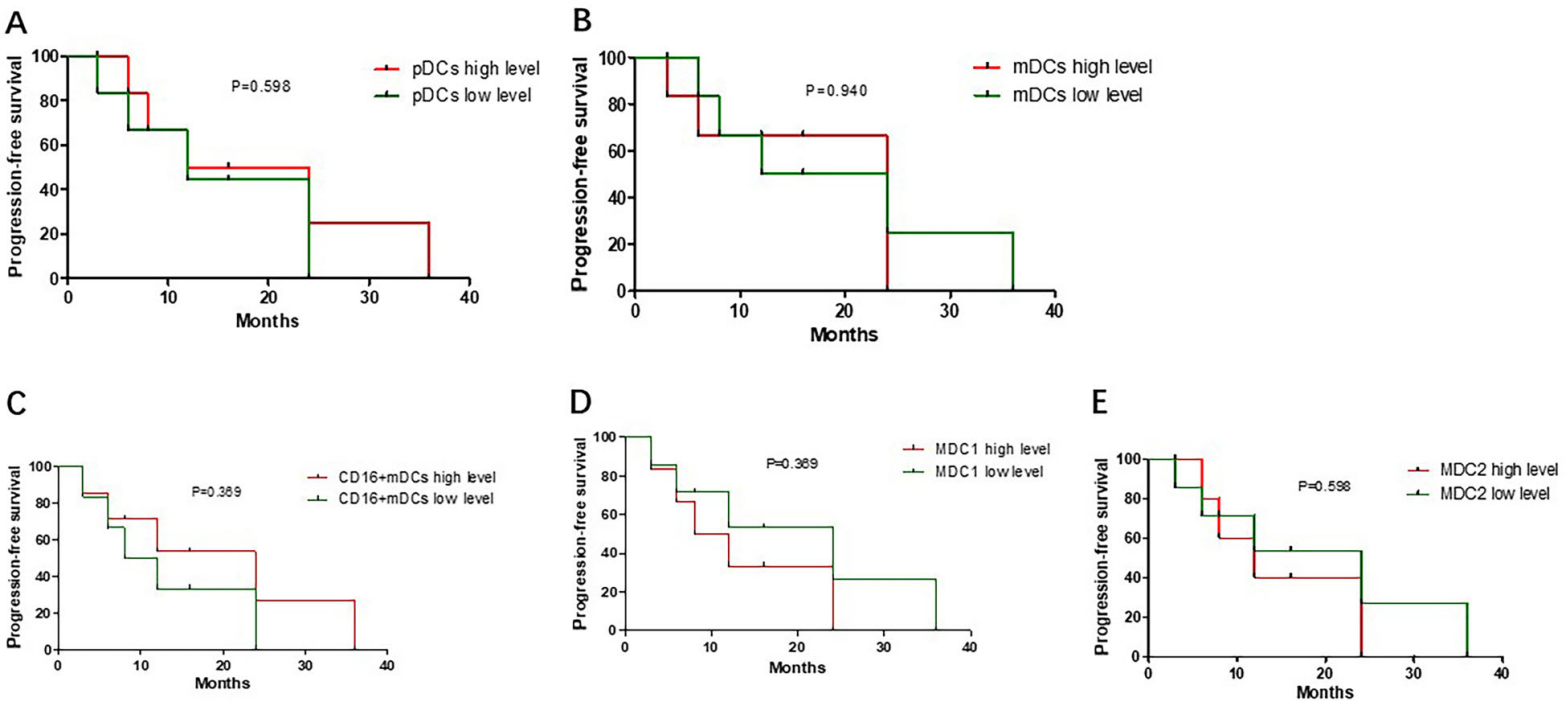
Progression-free survival analysis of subgroups divided by levels of plasmacytoid DC (pDCs) (**A**), myeloid DCs (mDCs) (**B**), CD16+mDCs (**C**), MDC1 (**D**), and MDC2 (**E**). Kaplan-Meier curves.

Enrolled patients with peritoneal adenocarcinoma were followed up as a cohort to assess tumor recurrence. Using flow cytometry, we investigated the association between circulating DC subpopulation and tumor PFS period in patients after the first surgery. Patients were divided into two groups based on the median percentage of DCs. The median values were: pDCs: 38.17%; mDCs: 60%; MDC1: 35.59%; CD16+mDCs: 59.66%; and MDC2: 0.63%. The results showed that there was no correlation between DC subsets and PFS (P>0.05, Figure 5A-E).

### Circulating DC subsets and their association with PD-1+T cells

**Figure 6:**
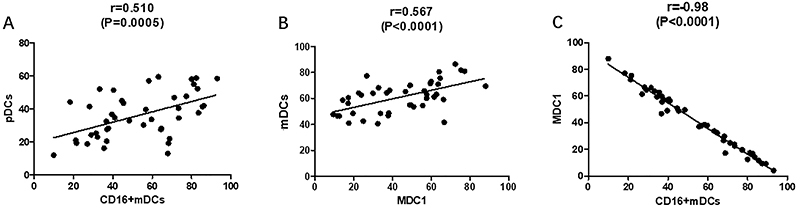
Correlations between percentage of dendritic cell (DCs) subsets (**A**) represented by percentage of plasmacytoid DC (pDCs) in peripheral blood and CD16+mDCs; **B**, between myeloid DCs (mDCs) and MDC1; and **C**, between CD16+mDCs and MDC1. P<0.05 was considered statistically significant. Pearson's correlation.

The percentage of circulating pDCs was positively correlated with CD16+mDCs subset (r=0.510, P=0.0005, Figure 6A), the same with mDCs and MDC1 (r=0.567, P<0.0001, Figure 6B). The frequencies of circulating MDC1 in peripheral blood were negatively correlated with CD16+mDCs (r=-0.980, P<0.0001, Figure 6C). All the results were analyzed under DCs.

**Figure 7:**
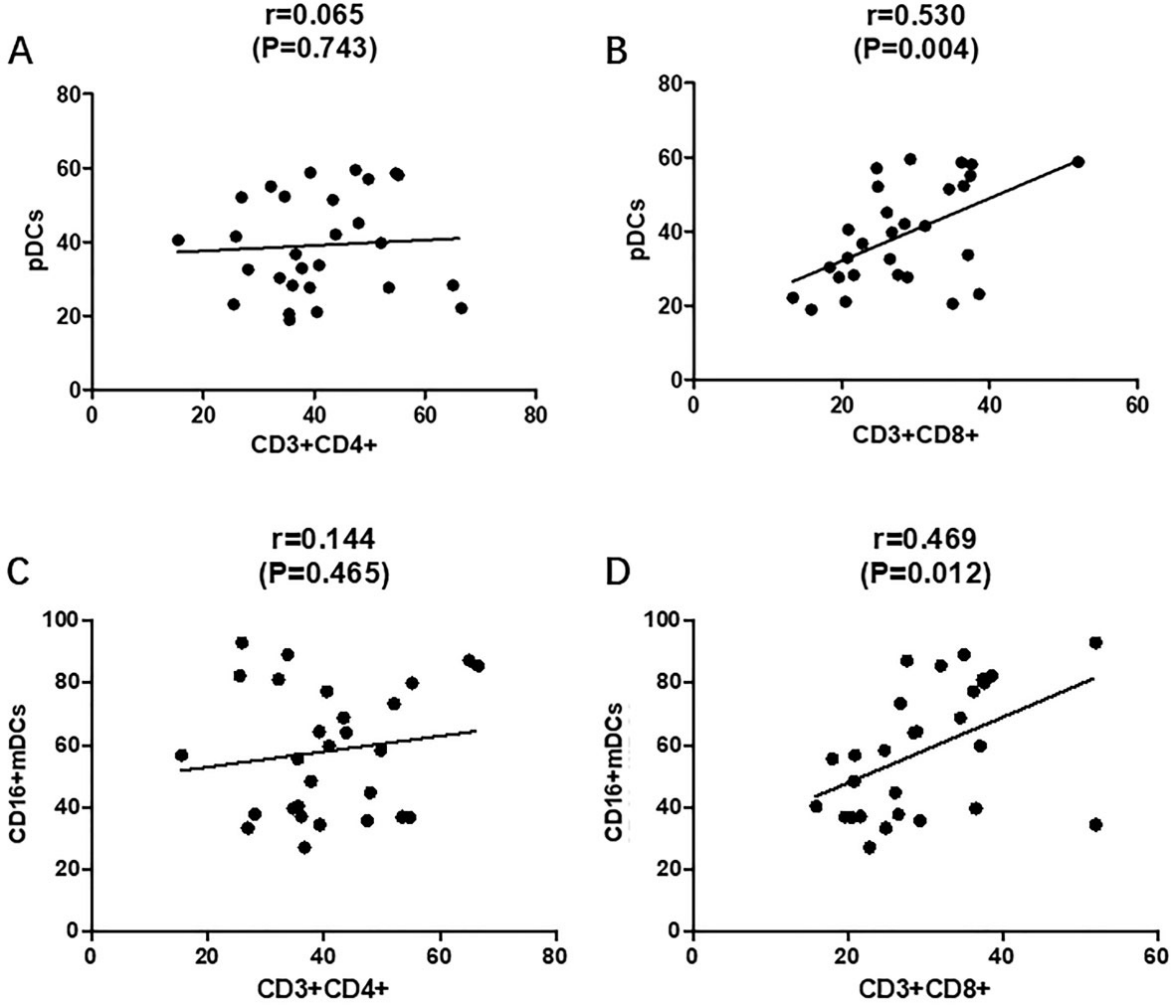
Correlations between plasmacytoid DC (pDCs), CD16+mDCs, CD3+CD4+T cells, and CD3+CD8+T cells. **A**, Correlation between pDCs and CD3+CD4+T cells; **B**, correlation between pDCs and CD3+CD8+T cells; **C**, correlation between CD16+mDCs and CD3+CD4+T cells; **D**, correlation between CD16+mDCs and CD3+CD8+T cells. mDCs: myeloid DCs. Pearson's correlation.

Compared with normal controls, pDCs and CD16+mDCs levels were higher in patients. In our previous reports, the percentages of CD4+T cells and CD8+T cells were higher in the peripheral blood of peritoneal adenocarcinoma patients. Therefore, the correlation between pDCs and CD16+mDCs, CD4+T cells, and CD8+T cells was explored. The percentages of pDCs and CD16+mDCs in DCs were positively correlated with CD3+CD8+T cells (r=0.530, P=0.004; r=0.469, P=0.0012, Figure 7B and D). The correlation between pDCs and CD16+mDCs and CD3+CD4+T cells had no statistical significance (r=0.065, P=0.743; r=0.144, P=0.465, Figure 7A and C).

**Figure 8:**
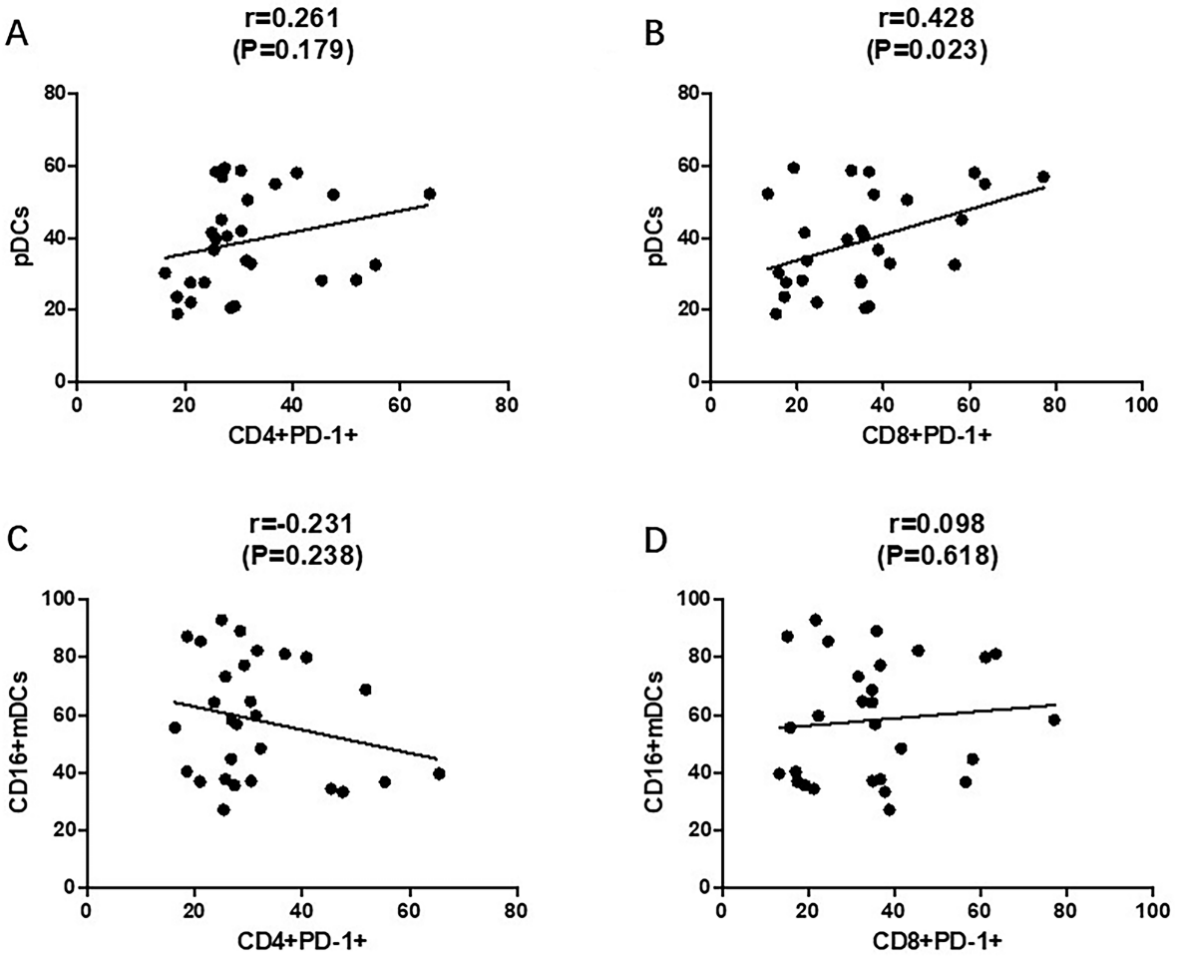
Correlations between plasmacytoid DC (pDCs), CD16+mDCs, CD4+PD-1+T cells, and CD8+PD-1+T cells. **A**, Correlation between pDCs and CD4+PD-1+T cells; **B**, correlation between pDCs and CD8+PD-1+T cells; **C**, correlation between CD16+mDCs and CD4+PD-1+T cells; **D**, correlation between CD16+mDCs and CD8+PD-1+T cells. mDC: myeloid DCs. Pearson's correlation.

The correlations between pDCs and CD16+mDCs, CD4+PD-1+T cells, and CD8+PD-1 T cells were also investigated. The percentage of pDC was positively correlated with CD8+PD-1+T cells (r=0.428, P=0.023, Figure 8B), and its correlation with CD4+PD-1+T cells had no statistical significances (r=0.261, P=0.179, Figure 8A). The levels of CD16+mDCs had no correlation with CD4+PD-1+T cells (r=0.231, P=0.238, Figure 8C) and CD8+PD-1+T cells (r=0.098, P=0.618, Figure 8D).

## Discussion

DCs are classified into different subsets according to the ontogeny, phenotype, tissue distribution, and molecular signatures both in mice and humans (25). According to reports, in the human body, tumors can secrete or stimulate other cells to secrete soluble factors to inhibit DCs. Studies have shown that IL-10, IL-6, and vascular endothelial growth factor (VEGF) produced by tumors can inhibit DC differentiation and function (26,27). IL-10, IL-6, and VEGF can upregulate transcription and metabolic pathways for DCs immune tolerance (28,29). Melanoma, multiple myeloma, and lung cancer can secrete IL-10 to inhibit the differentiation of monocytes into DCs and disrupt the antigen-presenting function of DCs (30,31). A recent study confirmed that increased serum IL-10 content in patients with liver cancer was associated with decreased DCs and phenotypic immaturity (32). These experiments suggest that in the tumor microenvironment, cytokines secreted by tumor cells inhibit the differentiation of CD34+ bone marrow stem cells into DCs, while negatively regulated DCs increase tumor aggressiveness (33).

In our report, the percentage of each cell subsets was analyzed under DC gate. Compared with normal controls, the frequencies of pDCs and CD16+mDCs were higher in the peripheral blood of peritoneal adenocarcinoma patients, while mDCs and its subsets (MDC1 and MDC2) presented opposite results. These results suggested that the percentage of DCs subsets in peripheral blood of tumor patients changed. We then explored the correlation between pDCs and CD16+mDCs, CD4+T cells, and CD8+T cells. The percentage of pDCs and CD16+mDCs in DCs were positively correlated with CD3+CD8+T cells, while the correlation between pDCs, CD16+mDCs, and CD4+T cells had no statistical significance. This may indicate the distinct roles of mDCs and pDCs in regulating T cell-mediated adaptive immunity. These correlations were also verified by Ito et al. (34), who found that the subsets of mDCs and pDCs have different expressions in T cells priming. As CD4+T cells, CD8+T cells also play an important role in the antigen presentation process of DCs.

One novel finding was the correlation between pDCs and CD8+PD-1+T cells. The blocking of PD-1 has been applied in the immunotherapy of many human cancers, such as melanoma, non-small-cell lung cell cancer, colorectal cancer, and bladder cancer (35-38). Our previous study (24) verified the assumption that in peripheral blood of peritoneal neoplasms patients, PD-1+T cells might participate in cancer development. Considering that DCs are the most efficient antigen-presenting cells, we further investigated the association between PD-1 and DCs subsets, which was positive. pDCs also positively correlated with CD8+PD-1+T cells, while the correlation between CD16+mDCs and CD8+PD-1+T cells had no statistical significance. These results indicate the heterogeneity and the different roles of DCs in the human cancer microenvironment.

According to reports, DCs are mainly involved in the induction and maintenance of immune tolerance in homeostatic conditions (39). In human peripheral blood, DCs constitute a rare cell population. This was why all the percentages of DCs subsets were analyzed under DC gate. The induction of an immune response is a very complex phenomenon, which involves interactions among different DCs. mDCs efficiently bind to CD3+CD8+T cells but activated CD3+CD8+T cells both recruit pDCs (40). DCs have the feature of cross-presentation, which allows DCs to prime against other cells' antigens. Our results showed that the percentage of pDCs and CD16+mDCs were positively correlated with CD3+CD8+T cells in peripheral blood of peritoneal adenocarcinoma patients. Patente et al. (39) report that the fate of T cells heavily depends on the interaction of DCs with T cells and the cytokines present in the microenvironment. This confirms that the antigen presentation process of DC cells *in vivo* is complex and the exact mechanisms are still unclear.

The present study had several limitations. First, further studies on the molecular mechanism of how DCs subsets cross-prime with other immune cells are needed. Second, this study had a small sample size, and a larger sample is needed to verify the correlation between DCs subsets and T cells and PD-1 in human peripheral blood. Thirdly, more in-depth experiments, such as functional studies, are needed to investigate the deeper correlation.

### Conclusion

In summary, DCs play an independent role in the immune function of peritoneal adenocarcinoma. The correlation between DCs and T cells and PD-1 can provide a new vision for clinical immunotherapy of peritoneal adenocarcinoma.
